# Diagnostic Algorithm in the Management of Acute Febrile Abdomen in Patients with Autosomal Dominant Polycystic Kidney Disease

**DOI:** 10.1371/journal.pone.0161277

**Published:** 2016-08-16

**Authors:** Marie Neuville, Roland Hustinx, Jessica Jacques, Jean-Marie Krzesinski, François Jouret

**Affiliations:** 1 Department of Nephrology, University of Liège Hospital (ULg CHU), Liège, Belgium; 2 Department of Nuclear Medicine, University of Liège Hospital (ULg CHU), Liège, Belgium; 3 Department of Medico-Economic Information, University of Liège Hospital (ULg CHU), Liège, Belgium; 4 Groupe Interdisciplinaire de Génoprotéomique Appliquée (GIGA), Cardiovascular Sciences, University of Liège, Liège, Belgium; Emory University Department of Medicine, UNITED STATES

## Abstract

**Background:**

Acute febrile abdomen represents a diagnostic challenge in patients with autosomal dominant polycystic kidney disease (ADPKD). Although criteria have been proposed for cyst infection (CyI) and hemorrhage (CyH), there is a lack of comparative assessments. Furthermore, distinguishing cystic from non-cystic complications remains problematic.

**Design:**

ADPKD patients presenting with abdominal pain and/or fever between 01/2005 and 06/2015 were retrospectively identified in a systematic computerized billing database. CyH was defined as spontaneous intracystic density above 50 Hounsfield units on computed tomography (CT). CyI was definite if confirmed by cyst puncture, and probable if 4 criteria were met: 3-day fever, loin/liver tenderness, C-reactive protein (CRP) plasma levels >50mg/L and no CT evidence for CyH. Other episodes were grouped as inflammation of unknown origin (IUO).

**Results:**

Among a cohort of 173 ADPKD patients, 101 presented with 205 episodes of abdominal pain (n = 172) and/or fever (n = 33). 20 patients experienced 30 CyH, whereas 16 presented 23 episodes of definite (n = 11) or probable (n = 12) CyI. 35 IUO were observed in 31 patients. Clinically, fever was observed in 7% *vs*. 100% *vs*. 66% of CyH, CyI and IUO, respectively. Biologically, CRP cut-off at 70 mg/dl showed 92% sensitivity and 81% specificity in CyI diagnosis. Urine or blood cultures remained sterile in >90% of CyH, but were contributive in 53.4% of CyI and IUO, with a 74.2% prevalence for *E*. *coli*. Radiologically, ultrasounds, CT and magnetic resonance diagnosed CyI in 2.6%, 20% and 16.7% of cases, respectively. ^18^F-FDG positron-emission tomography (PET)/CT was done within a median period of 7 days *post* antibiotics, and significantly changed patient management in 71.4%.

**Conclusions:**

This retrospective single-center series underscores the usefulness of clinical–fever–and biological–CRP–parameters, but emphasizes the limitations of bacteriological and radiological investigations in cases of acute febrile abdomen in ADPKD patients. ^18^F-FDG-PET/CT imaging may be helpful in such condition.

## Introduction

Autosomal dominant polycystic kidney disease (ADPKD) is a common inherited disorder characterized by the development of cysts in the renal parenchyma irreversibly leading to chronic kidney disease (CKD) [1,2]. ADPKD is the fourth most common cause for renal replacement therapy [1]. Extra-renal manifestations, including hepatic cysts and connective tissue abnormalities, are frequently observed in ADPKD patients [1,3,4].

Acute cyst complications, including cyst hemorrhage (CyH) and infection (CyI), represent severe conditions of ADPKD. The incidence of CyI has been reported as 0.01 episode per patient per year [5]. CyI has been associated with substantial risk for abscess formation and life-threatening sepsis, which necessarily requires early and appropriate management [6]. The diagnostic challenge of CyI may delay and/or cause erroneous patients’ management [7–9]. Furthermore, discriminating acute cyst complications from non-cystic abdominal diseases is often difficult on the basis of unspecific clinical, biological and radiological parameters [8,10,11]. Kidney and liver functions are usually unchanged at the time of CyH or CyI, and blood or urine cultures most often remain sterile. Similarly, conventional imaging methods, namely ultrasounds (US) and computed tomography (CT), show poor diagnostic yield in CyI, even after administration of CT contrast agent [7,8,10].

Over the past decade, diagnostic criteria for CyH and CyI have been proposed [5,7,10,12–14], as recently summarized by Lantinga M.A. and colleagues [8]. Typically, CyH is associated with spontaneous intracystic density above 50 Hounsfield units on CT. CyI is “definite” when confirmed by cyst content analysis showing neutrophils or bacteria. However, cyst puncture may provoke rupture or contamination of adjacent cysts, and is therefore rarely performed. Hence, CyI is regarded as “probable” when 4 criteria are concomitantly met: 3-day fever >38°C, loin or liver tenderness, C-reactive protein (CRP) plasma levels >50mg/L, and no CT evidence for CyH [5,10]. Additional clinical criteria, like weight loss or recent instrumentation of urinary or biliary tract, and biological parameters, like hematuria or white blood cell (WBC) count, have been listed, although their respective diagnostic yield remains unclear [8]. Isolated reports suggest that diffusion sequences in magnetic resonance imaging (MRI) help improve both sensitivity and specificity in CyI diagnosis[10,15]. Finally, 18-Fluoro-deoxy-glucose (^18^FDG) positron-emission tomography (PET) has recently proven useful in infectious diseases, including CyI [5,7,10,11,16–18].

In the present retrospective single-center series, we first characterized the monocentric incidence of acute cyst complications in ADPKD patients over a 10-year period. Next, we investigated the diagnostic yield of clinical, biological and imaging parameters in distinguishing CyI from CyH and non-cystic conditions. Finally, we propose a diagnostic algorithm for the management of ADPKD patients presenting with acute febrile abdomen.

## Patients and Methods

### Patients

This study was approved (#2016/108) by the Commission of Biomedical Ethics of the University of Liège Hospital (ULg CHU) in Liège, Belgium. Because of the retrospective design of the study, no informed consent was given by the patients. Still, data were anonymised and de-identified prior access and analysis. Using the systematic computerized billing database of ULg CHU, ADPKD patients presenting with abdominal pain and/or fever between January 2005 and June 2015 were retrospectively identified. The keywords were: “polycystic liver/congenital” or “polycystic kidney, autosomal dominant” for all adult patients admitted to ULg Academic Hospital (ULg CHU), including the emergency room. This search retrieved 234 patients. Next, all medical files were systematically reviewed to exclude patients in whom conventional criteria for ADPKD diagnosis proposed by Pei et al. were not met [19]. This systematic approach led to the exclusion of 61 patients.

### Diagnostic criteria for cyst complications

The diagnosis of CyH and CyI were based on the criteria proposed by Sallée M. and colleagues[5]. Inflammation of unknown origin (IUO) included all episodes in which diagnostic criteria for CyH or CyI were not met, in the absence of evidence for a non-cystic disease.

### Imaging criteria for cyst infection

US, CT and MRI were considered as positive for CyI when enhanced wall thickening and inflammatory infiltrates were detected in at least one cyst. More particularly, diagnostic MRI criteria also included significant diminishment in diffusion on diffusion-weighted sequences. ^18^FDG-PET/CT was considered as positive for CyI when ^18^FDG uptake was increased around at least one cyst[17].

### Statistics

Continuous variables were checked for normality using the Kolmogorov-Smirnov test, and homogeneity of variance was assessed using the Bartlett’s test. Non-parametric approach was used when data did not satisfy the tests assumptions. Means were compared between groups using Student t test or one way ANOVA with F test. Mann Withney U test or Kruskal Wallis teste were used otherwise. Categorical values are reported as number and percentages, and compared with chi square test or Fisher exact test. A p-value below 0.05 was considered as significant. Analyses were performed using Graphpad Prism 5.0.

## Results

### Cohort of patients with ADPKD presenting with suspected acute cyst complication

**Fig 1:**
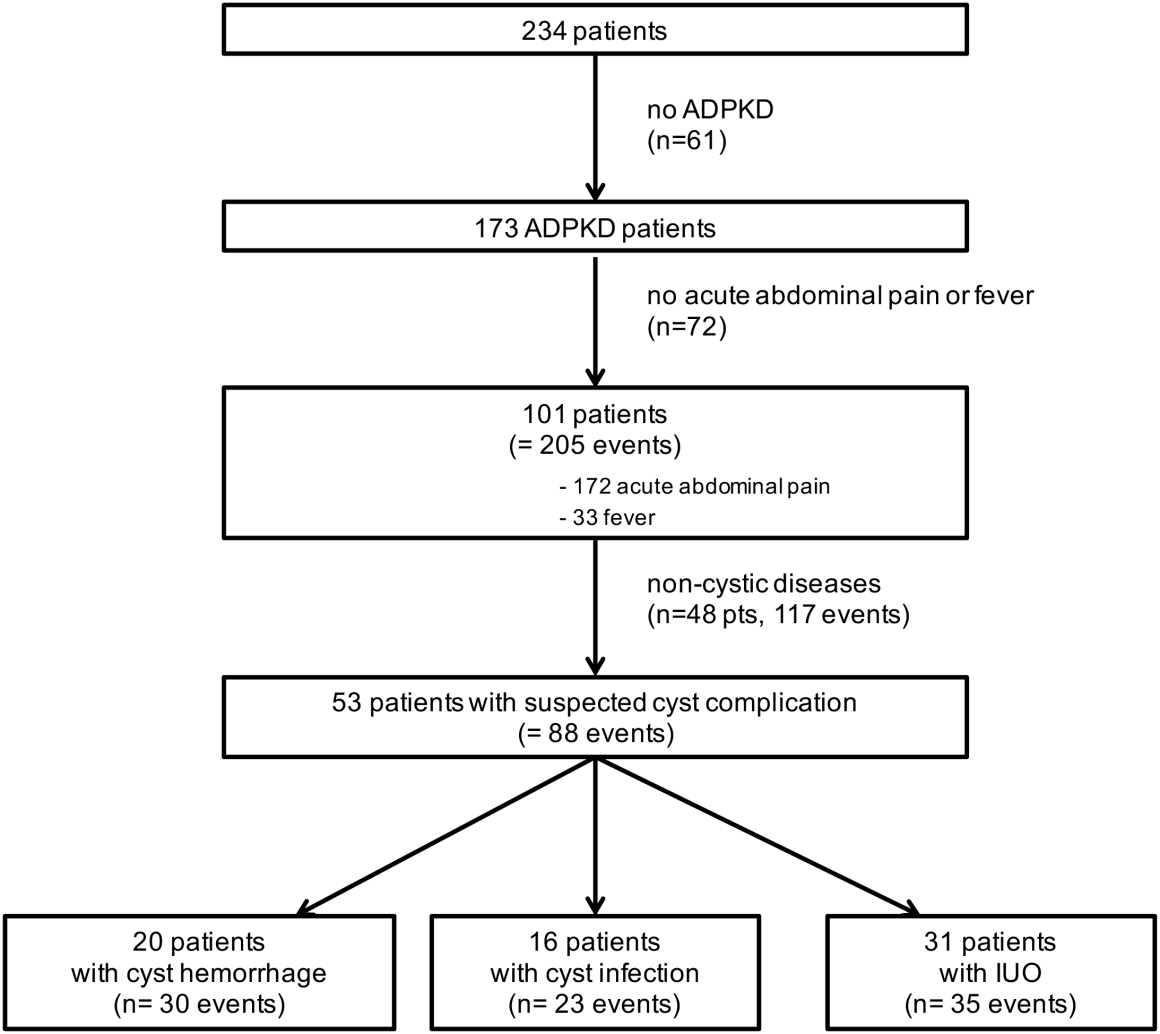
Selection process of patients with autosomal dominant polycystic kidney disease (ADPKD) presenting with suspected acute cyst complication. One given patient may present with different types of cyst complications

We identified 173 patients with ADPKD who were admitted between 2005 and 2015 (Fig 1). Among these, 101 patients (58.4%) presented with 205 events of acute abdominal pain (n = 172) and/or fever (n = 33). In 48 ADPKD patients, i.e. 117 events, diagnostic work-up using urine and blood analysis, as well as US or CT, detected non-cystic diseases, including colorectal (n = 27, with 7 diverticulitis and 7 *Clostridium difficile* colitis), urologic (n = 27, with 7 kidney stones and 6 prostatitis), upper gastrointestinal (n = 11), bronco-pulmonary (n = 11, with 9 pneumonia), iatrogenic (n = 8, with 6 post-surgical complications), hepato-pancreatic (n = 7) and viral diseases (n = 7, with 4 CMV infections), as well as peritonitis (n = 6), neoplasia (n = 2), and others (n = 11). Of note, bacteremia is a sign of infection, but does not provide the exact origin of the infection. Similarly, urosepsis in ADPKD patients may originate from both cystic and non-cystic infection. So, our series included ADPKD patients with suspected cyst complication, including documented urosepsis (by positive urinoculture) with no clear diagnostic evidence for a renal or a non-renal origin.

In 53 ADPKD patients, 88 events of acute cyst complications were suspected (S1 File). One given patient may present with different types of cyst complications (S2 File). This cohort included 7 episodes occurring in patients under chronic dialysis and 39 episodes occuring in kidney transplant recipients (KTR). Twenty patients showed a total of 30 episodes of CyH, as confirmed by CT, and 16 patients presented with 23 episodes of CyI (Fig 1). Among these, 7 and 4 cases were definite kidney or liver CyI, respectively, and 10 and 2 were probable kidney or liver CyI, respectively. Thirty-five episodes of suspected cyst complications in 31 patients were categorized as IUO (Fig 1). As a whole, CyH and CyI in patients with ADPKD represented 25.8% of hospital admissions for abdominal pain and/or fever. Of important note, no death could be directly attributed to CyH or CyI.

### Clinical and biological parameters

Table 1 summarizes pertinent parameters observed in 88 episodes of suspected acute cyst complication. Cases of definite CyI included 6 surgical procedures of nephrectomy (n = 5) or partial hepatectomy (n = 1), and 5 percutaneous cyst punctures. Bacteriological identification in cyst fluid was successful in 3 cases (27.3%), whereas 8 episodes showed neutrophils in cyst lumen. Clinically, abdominal pain was found in nearly all patients with CyH or definite CyI. Fever was observed in all patients with definite CyI, but was virtually absent in patients with CyH (n = 2/30). Biologically, no significant difference was found between renal CyH (22.2%) *versus* renal CyI (35.3%) regarding hematuria (Table 1). By contrast, leucocyturia was detected significantly more frequently in renal CyI than renal CyH (41.1% *vs*. 11.1%, *p<0*.*05*). Groups of CyH and definite CyI showed significant differences in CRP values at hospital admission (13.3±14.2 *vs*. 187.3±95.7, *p<0*.*001*) and at peak within the first 48h *post* admission (35.2±58.9 *vs*. 229.1±105.1, *p<0*.*001*). The area under the receiver operating characteristic (ROC) curve (AUC) for CRP levels in CyI diagnosis reached 0.91 for a cut-off set at 70 mg/dl, with a sensitivity of 92% and a specificity of 81% (S1 Fig). By contrast, ANOVA showed similar levels of WBC count at the admission (*p*, *0*.*20)* and within the first 48h *post* admission (*p*, *0*.*14*) in all groups of CyH, probable and definite CyI or IUO (Table 1).

**Table 1:** Clinical and biological characteristics of the cohort.

	n	Age	Gender	Dialysis	KTR	eGFR*	T°>38°C	Pain	WBC	CRP	Hematuria	Leucocyturia	Germ ID		PET/CT
		(years)	(male, %)	(%)	(%)	(ml/min)	(%)	(%)	(10^6^/mm³)	(mg/L)	(%)	(%)	Urine (%)	Blood (%)	(+, %)
**Hemorrhage**	30	46 ± 13	50	0	17	77 ± 49	7	97	9.7 ± 3.3	13 ± 14	20	10	10	7	0/1, 0
**Renal cyst infection**															
**Definite**	7	52 ± 11	70	15	43	39 ± 16	100	86	11.3 ± 3.9	163 ± 98	29	43	15	57	3/3, 100
**Probable**	10	48 ± 14	30	20	30	61 ± 43	100	100	10.8 ± 3.1	252 ± 204	40	40	40	20	3/4, 75
**Liver cyst infection**															
**Definite**	4	63 ± 5	50	25	75	29 ± 14	100	100	12.1 ± 2.6	230 ± 87				50	1/1, 100
**Probable**	2	[66; 66]	50	50	50	38	100	100	[4.2; 6.5]	[51; 342]				50	1/2, 50
**IUO**	35	55 ± 15	50	6	66	49 ± 31	66	66	11.8 ± 5.6	98 ± 92	37	60	48	23	7/17, 42

KTR, kidney transplant recipients; eGFR, estimated glomerular filtration rate; WBC, white blood cells at admission; CRP, C-reactive protein level at admission; ID, identification; IUO, inflammation of unknown origin. Mean +/- Standard Deviation.

### Microbiological documentation

Among 88 episodes of suspected acute cyst complications in 53 ADPKD patients, a pathogen bacterium was identified in 34 cases (38.6%), either in urine (n = 25, 28.4%), in blood (n = 19, 21.6%), or in both (n = 10, 11.4%) (Table 2). The most common organism was *Escherichia coli* (70.6%), followed by *K*. *pneumoniae* (11.8%). Focusing on CyI, the causative bacterium was found in 12 cases (52.2%), either in urine (n = 5, 21.7%), in blood (n = 9, 39.1%) or in both (n = 2, 8.7%). *E*. *coli* represents the most frequent pathogen, with a prevalence of 91.7%. In IUO, causative pathogens were identified in 19 cases (54.3%), either in urine (n = 17, 48.6%), in blood (n = 8, 22.9%) or in both (n = 6, 17.1%). Expectedly, urine and blood cultures were rarely positive (<10%) in CyH. Furthermore, contamination at the time of collection cannot be excluded given the types of the organism, including *Staphylococcus epidermidis* or *Streptococcus agalactiae*.

**Table 2:** Bacteriological documentation.

	n	Pathogen ID (%)	Blood (+, %)	Blood Strains	Urine (+, %)	Urine Strains
**Hemorrhage**	30	10	7	*S*.*epidermidis (n = 1)*	10	*S*. *agalactiae (n = 1)*
				*K*. *pneumoniae (n = 1)*		*E*. *coli (n = 1)*
**Renal cyst infection**						
**Definite**	7	60	57	*E*. *coli (n = 2)*	15	*E*. *coli (n = 1)*
				*S*. *epidermidis (n = 1)*		
				*S*. *capitis (n = 1)*		
**Probable**	10	50	20	*E*. *coli (n = 2)*	40	*E*. *coli (n = 4)*
**Liver cyst infection**						
**Definite**	4	50	50	*E*. *coli (n = 2)*	0	
**Probable**	2	50	50	*Ecoli (n = 1)*	0	
**IUO**	35	55	23	*E*.*coli (n = 4)*	50	*E*. *coli (n = 11)*
				*K*. *pneumoniae (n = 2)*		*K*. *pneumoniae (n = 2)*
				*S*. *epidermidis (n = 1)*		*S*. *aureus (n = 1)*
				*S*. *aureus (n = 1)*		*E*. *faecalis (n = 1)*
						*C*. *koseri (n = 1)*
						*P*. *mirabilis (n = 1)*

### Conventional imaging techniques

Among 58 episodes of suspected cyst complications with no radiological evidence of CyH, 38 abdomen US were performed (67.9%), among which only 2 were suggestive of CyI. Likewise, 35 CT were performed (60.3%), including 13 after administration of contrast agent. CyI was diagnosed in 7 cases (20%), among which 4 were performed using radiological agent. Finally, 6 MRI were performed (10.4%), with one contributive exam.

### ^18^FDG-PET/CT imaging

**Fig 2:**
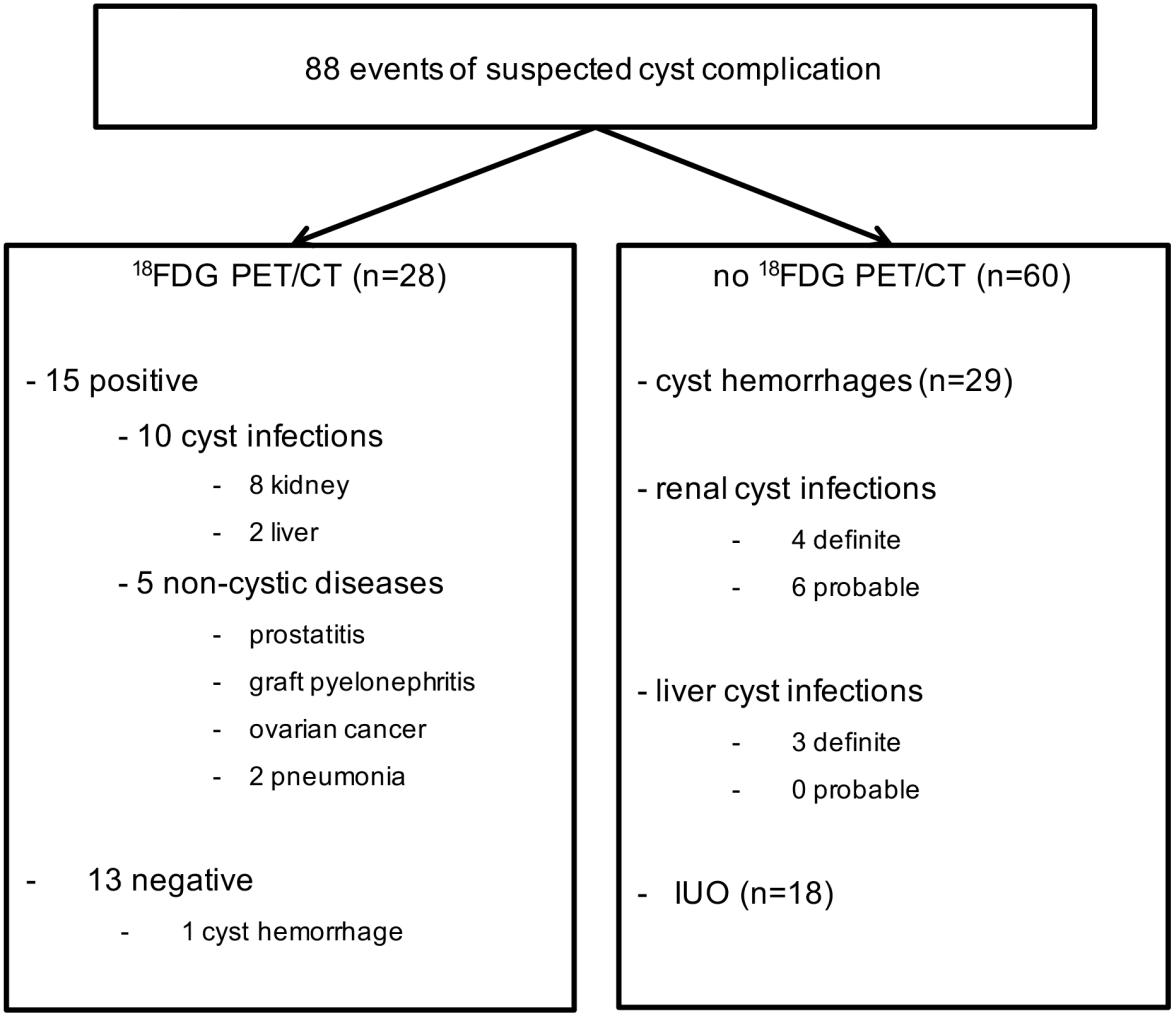
Distribution of ^18^FDG-PET/CT imaging in patients with autosomal dominant polycystic kidney disease (ADPKD) presenting with suspected cyst complication. The final diagnosis is provided on the basis of the entire work-up.

^18^FDG-PET/CT was performed in 28 cases (31.8%) of suspected cyst complication (Fig 2) within a median period of 11 days [3d; 28d] following admission and a median period of 7 days [0d; 30d] following the initiation of antibiotics. Of note, ^18^FDG-PET/CT imaging in our series was particularly used in ADPKD KTR (20/28, 71.4%). Among 11 definite CyI, 4 ^18^FDG-PET/CT were performed and confirmed the diagnosis in all cases (Table 1).

Among 12 probable CyI, ^18^FDG-PET/CT confirmed the diagnosis in 4/6 cases. The first patient with probable CyI and negative ^18^FDG-PET/CT was a 66-year-old female KTR who had been admitted for fever, myalgia and right hypochondrium pain for 15 days. She had previously benefited from bilateral nephrectomy. Blood analysis showed CRP and WBC levels at 50.6 mg/L and 4.250/mm^3^, respectively. Urinalysis did not detect hematuria or leucocyturia, and remained sterile. CT was not contributive, nor was ^18^FDG-PET/CT performed 4 weeks *post* admission. No antibiotic therapy was initiated, and the patient spontaneously recovered. The second patient with probable CyI and negative ^18^FDG-PET/CT was a 65-year-old woman who presented with fever and right flank pain for 3 days. Blood analysis revealed CRP and WBC levels at 463.2 mg/L and 12.240/mm^3^, respectively. Urinalysis detected hematuria without leucocyturia. Urine and blood cultures remained sterile. CT was not contributive. Still, i.v. antibiotic therapy was administered for 14 days, with a sharp and significant recovery. ^18^FDG-PET/CT was performed 6 days after antibiotherapy ended. Follow-up was unremarkable.

Finally, ^18^FDG-PET/CT was performed in 17 IUO, with 2 of them (11.7%) highlighting renal CyI. The first case was a 64-year-old female KTR admitted for isolated inflammatory syndrome detected 15 days following kidney transplantation. Blood analysis showed CRP and WBC levels at 214 mg/L and 13.400/mm^3^, respectively. Urinalysis detected leucocyturia without hematuria. Urine and blood cultures remained sterile. US of native and transplanted kidneys was not contributive. Still, ciprofloxacine therapy was initiated. ^18^FDG-PET/CT was performed 1 week *post* treatment initiation, and showed renal CyI. Antibiotics were maintained for 6 weeks, and the evolution was favorable. The second case was a 46-year-old male KTR who presented with fever, dysuria and pollakiuria for 2 days. His general practitioner immediately started cefuroxime. Blood analysis at admission revealed CRP and WBC levels at 250 mg/L and 12.470/mm^3^, respectively. Urine and blood cultures remained sterile. Abdominal US was not contributive. ^18^FDG-PET/CT was performed at day 13 after cefuroxime initiation, which was subsequently shifted to ciprofloxacin for 6 weeks given PET/CT results suggestive of CyI. In addition to these 2 CyI, 5 non-cystic inflammatory diseases were identified by ^18^FDG-PET/CT among IUO, including prostatitis, graft pyelonephritis, ovarian cancer and pneumonia (n = 2) (Fig 2). Of note, 1 ^18^FDG-PET/CT was performed among 30 episodes of CyH (3.3%), and did not show any increase in renal ^18^F-FDG uptake.

In sum, among 27 ^18^FDG-PET/CT performed in 58 events of suspected cyst complications excluding CyH, 10 (37%) led to the diagnosis of renal (n = 8) or hepatic (n = 2) CyI, 5 (18.5%) showed non-cystic diseases and 12 (44.4%) were not contributive (Table 3). Among these 12 negative ^18^FDG-PET/CT, 4 were done in ADPKD patients who spontaneously recover, which reasonably excludes CyI. By contrast, the remaining 8 negative ^18^FDG-PET/CT were done following a median period of 11 days [3d; 20d] of antibiotics. On the basis of ^18^FDG-PET/CT negative results, the duration of antibiotic therapy was reduced in 5/8 cases (62.5%) to a median period of 18 days [14d; 21d]. As a whole, the management of the patient was significantly changed by ^18^FDG-PET/CT results in 20 cases (71.4%). More specifically, negative ^18^FDG-PET/CT (n = 13) lead to significant modifications of therapeutics in 7 cases (53.8%). Positive ^18^FDG-PET/CT detecting a non-cystic inflammation changed patient management in all cases (n = 5, 100%). Positive ^18^FDG-PET/CT detecting cyst infection (n = 10) significantly modified therapeutics, including the duration of antibiotics, in 8 cases (80%).

**Table 3:** Clinical and biological characteristics of the cohort upon ^18^FDG PET/CT use.

	n	Age	Gender	Dialysis	KTR	eGFR	WBC	CRP	Germ ID	
									Urine	Blood
		(years)	(male, %)	(%)	(%)	(ml/min)	(10^6^/mm³)	(mg/L)	(+, %)	(+, %)
^**18**^**FDG PET/CT**	28	56 ± 11	57	14	71	40 ± 17	10.2 ± 3.7	127 ± 125	28	28
**cyst infection**	10	52 ± 11	70	20	50	36 ± 15	10.2 ± 2.4	207 ± 120	10	30
**non-cystic diseases**	5	60 ± 10	60	0	100	40 ± 23	10.5 ± 2.6	38 ± 12	60	40
**no** ^**18**^**FDG uptake**	13	56 ± 11	46	15	77	44 ± 17	10.1 ± 4.9	99 ± 121	31	23
**No** ^**18**^**FDG PET/CT**	60	49 ± 15	50	5	31	67 ± 45	11.1 ± 4.7	89 ± 125	28	18
**excl. cyst hemorrhage**	31	53 ± 17	42	10	48	50 ± 38	12.4 ± 5.5	157 ± 141	45	29

KTR, kidney transplant recipients; eGFR, estimated glomerular filtration rate; WBC, white blood cells at admission; CRP, C-reactive protein level at admission; ID, identification. Mean +/- Standard Deviation.

## Discussion

Acute cyst complications, including CyH and CyI, are common manifestations of ADPKD. At Liège Academic Hospital (ULg CHU), CyH and CyI represent 25.8% of hospital admissions for abdominal pain and/or fever in ADPKD patients. There is an established association between renal cyst complications and the slope of CKD progression [20]. Hence, an urologic event before the age of 35 has been recently proven as an independent risk factor for CKD progression[21–23]. In case of suspected cyst complication in ADPKD patients, the main diagnostic steps include (i) to rule out non-cystic pathologies, (ii) to discriminate CyI from CyH, and (iii) to exclude concurrent conditions, such as urinary or biliary tract obstruction[10]. Treatment of CyI ideally requires localizing pyocysts and identifying causative organisms. Even though such a sequence appears obvious, the real-life management of cyst complication is complex because of the lack of specific symptoms, signs and imageries distinguishing cystic from non-cystic diseases [10]. Consequently, patients may be exposed to delayed or erroneous treatment. Indeed, while CyH treatment most often relies on painkillers and expectant management, CyI requires an early and prolonged antibiotherapy, with inherent exposure to side-effects and cost [11,24]. Furthermore, liver pyocyst should be drained in case of resistance to well-conducted antibiotherapy and/or rapid relapse[25,26]. Mortality is mostly linked to liver CyI [13,25].

The design of the present study mimics “real-life” situations, including all APDKD patients presenting with suspected cyst complication. Clinically, pain did not help discriminate CyI from CyH, whereas fever was significantly more frequent in CyI than CyH. Biologically, CRP levels were significantly higher in CyI than CyH. Hence, the AUC for CRP levels in CyI diagnosis reached 0.91 for a cut-off set at 70 mg/dl, with a sensitivity of 92% and a specificity of 81%. No difference was observed among groups concerning WBC counts. In urinalysis, hematuria was observed in both renal CyI and CyH, whereas leucocyturia was mostly found in renal CyI. In line with previous observations, pathogens were identified only in ~50% of CyI. Such an information is essential for tailoring antibiotics, but does not reliably distinguish cystic from non-cystic infections[8,10]. Radiologically, abdomen US, CT and MRI diagnosed CyI in 2.6%, 20% and 16.7% of cases, respectively. Abdomen US was performed in 47% of ADPKD patients with CyI, with a positive yield of 9%. Such a low sensitivity questions its clinical usefulness in assessing an ADPKD patient with suspected cyst complication. Ninety-one % of CyI underwent CT. The main limitation of CT in ADPKD patients with CKD is the contra-indication for i.v. contrast medium. In our series, 57% of CT were performed with contrast agent. Only 33% and 22% of CT with or without contrast infusion disclosed CyI. Such limited gain observed after administration of contrast material should be individually gauged on the basis of patient’s baseline renal function. Conversely, CT imaging was useful to rule out non-cystic diseases in 43 cases among 117 events (Fig 1) and CyH. Finally, abdomen MRI with (n = 2) or without (n = 2) diffusion-weighed imaging was performed in 17% of CyI, with a contributive yield of 25%. Because of its cost, restricted availability and poor diagnostic yield, the role of MRI remains unclear in the management of ADPKD patients with suspected cyst complication.

Accumulative evidence supports that ^18^FDG-PET/CT may help (i) diagnose CyI, (ii) discriminate CyI from non-cystic disease and (iii) localize pyocysts, thereby facilitating the management[17,27–29]. Of note, CyH has not been reported thus far with pathological uptake of ^18^FDG, which further help distinguish CyI from CyH[11,17]. The advantages of ^18^FDG-PET/CT are rapid imaging, high target-to-background ratio, direct co-registration with low-dose CT, and the absence of toxicity[17,30,31]. Limitations include cost and availability. In addition, ^18^FDG uptake is not specific to infection[31,32]. In Bobot M. et al.[11], ^18^FDG-PET-CT showed 77% sensitivity and 100% specificity for CyI diagnosis. In our series, ^18^FDG-PET/CT was performed in 10/23 CyI, with a positive yield of 8/10. One plausible explanation for negative results is that the procedure was performed later than the average. The impact of antibiotics on ^18^FDG-PET/CT results is debated. Bobot M. et al. recommend ^18^FDG-PET/CT within 7 days of antibiotics initiation in ADPKD patients with suspected CyI[11,27]. Lantinga M. and colleagues propose that ^18^FDG-PET/CT might help monitor antibiotics efficiency, which indirectly suggests that late imaging induces false negative results. By contrast, observations of Piccoli G.B. and colleagues support that ^18^F-FDG accumulation around pyocysts remains detectable up to 6 weeks *post* treatment[7].

**Fig 3:**
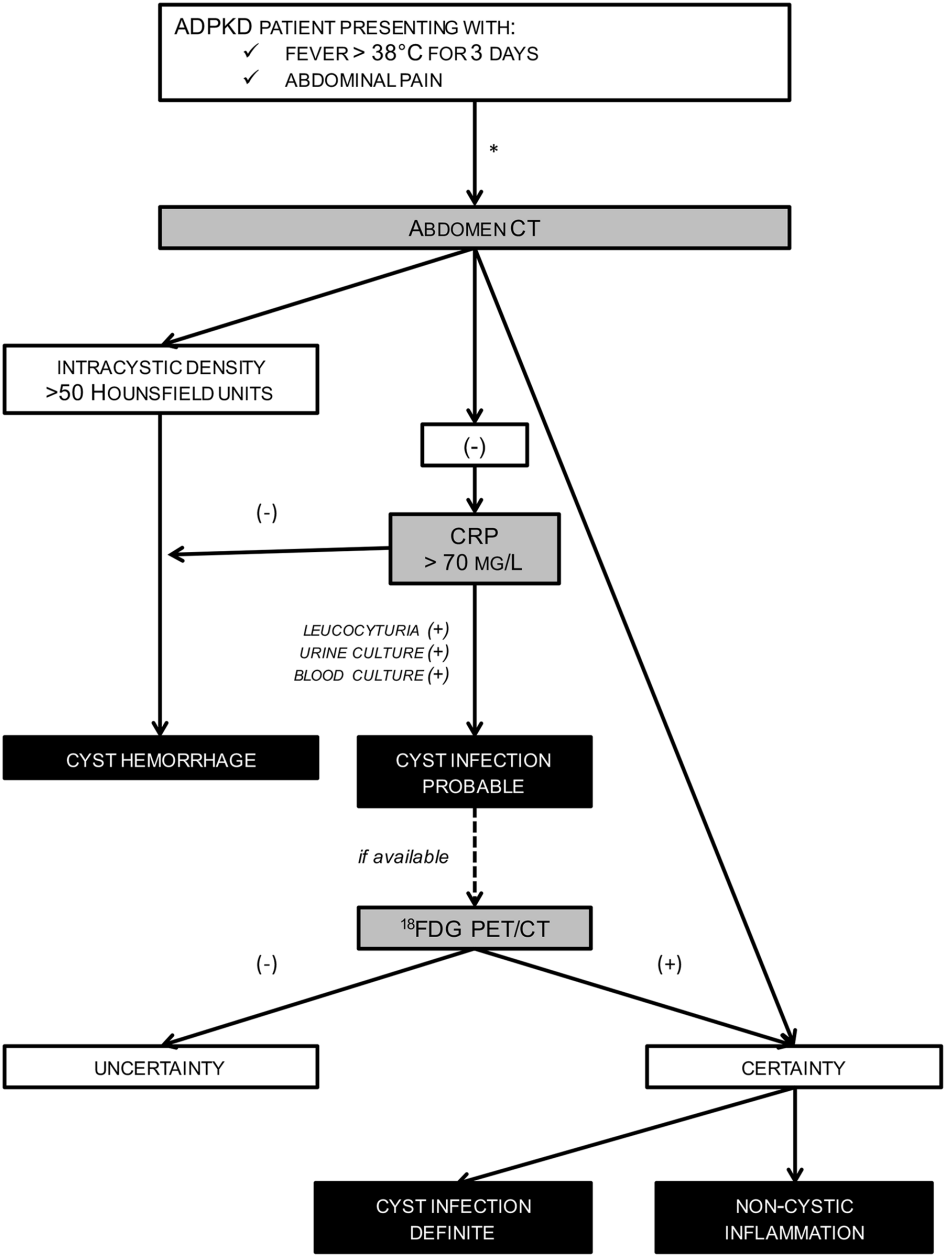
Diagnostic algorithm to manage patients with autosomal dominant polycystic kidney disease (ADPKD) presenting with suspected acute cyst complication. *, On the basis of the conventional management (including blood and urine analyses and abdomen imaging) of any patient with febrile abdominal pain in emergency conditions (33).

On the basis of these observations, we propose an algorithm to help clinicians discriminate CyI from CyH and non-cystic diseases in ADPKD patients presenting with suspected acute cyst complication (Fig 3). This is grounded on the conventional management of any patient with acute febrile abdominal complaint [33], and starts with 3 simultaneous conditions: ADPKD, fever (>38°C for 3days) and abdominal pain. If such a constellation of 3 criteria is not met, the management of patients may follow another algorithmic approach, which is out of scope of the present study. The first steps rely on blood analyses, including CRP plasma levels, and abdomen CT. The use of contrast agent should be restricted to ADPKD patients with preserved kidney function. CT will help categorize the patients into 3 groups: (i) CT-proven CyH, (ii) CT-proven cystic or non-cystic disease, and (iii) non-contributive CT. Such crucial information would significantly change the management of ADPKD patients with fever and abdominal pain. Next, threshold of CRP is set at 70 mg/L. CyH is most likely in case of low CRP, as reported by us and others[5,7,8,10]. By contrast, CRP above 70 mg/L is highly suggestive of CyI. Urinalysis-proven leucocyturia would further support the diagnosis of renal CyI. Blood and urine cultures should be executed before treatment initiation to eventually tailor antibiotic therapy. On the basis of these criteria initially proposed by Sallée M. and colleagues[5], diagnosing CyH or probable CyI is achievable, thereby allowing early and appropriate management. In order to (i) confirm CyI diagnosis, (ii) locate pyocysts and (iii) eventually monitor therapeutic efficiency, ^18^FDG-PET/CT should be performed within 7 days *post* antibiotics.

Several limitations of this study have to be acknowledged, including the retrospective monocentric design with a limited number of heterogeneous patients, including CKD stages, ESRD under chronic hemodialysis and KTR. The decision to perform imaging procedures, including US, CT, MRI and/or ^18^F-FDG-PET/CT, was at the discretion of physicians in charge in the absence of pre-established criteria. Few cyst punctures were performed, which weakens the diagnostic gold-standard. However, the “real-life” design encompassing all ADPKD patients with abdominal pain and/or fever allowed us to compare clinical, biological and imaging parameters in CyI *versus* CyH and non-cystic diseases, thereby proposing a diagnostic algorithm. Prospective trials are needed to test the significance of this algorithm, as well as to better delineate the timing of ^18^FDG-PET-CT in diagnosis (and follow-up) of ADPKD patients with acute febrile abdomen.

## Supporting Information

S1 Fig Receiver operating characteristic (ROC) curve of CRP plasma levels for the diagnosis of cyst infection in 88 episodes of acute febrile abdomen in 53 patients with autosomal dominant polycystic kidney disease (ADPKD). The area under the curve reaches 0.91 for a cut-off set at 70 mg/L, with a sensitivity of 92% and a specificity of 81%.
(TIF)

S1 File Clinical characteristics of 88 episodes of suspected acute cyst complication in 53 patients with autosomal dominant polycystic kidney disease (ADPKD). (PDF)

S2 File Detailed characteristics of the cohort including 53 patients with autosomal dominant polycystic kidney disease (ADPKD) presenting with 88 episodes of suspected acute cyst complication. (PDF)
