# Supplementary figures and images for: Diagnostic Algorithm in the Management of Acute Febrile Abdomen in Patients with Autosomal Dominant Polycystic Kidney Disease
Source: PLoS One. 2016 Aug 16;11(8):e0161277. doi: 10.1371/journal.pone.0161277 (PMC4987061; doi:10.1371/journal.pone.0161277)

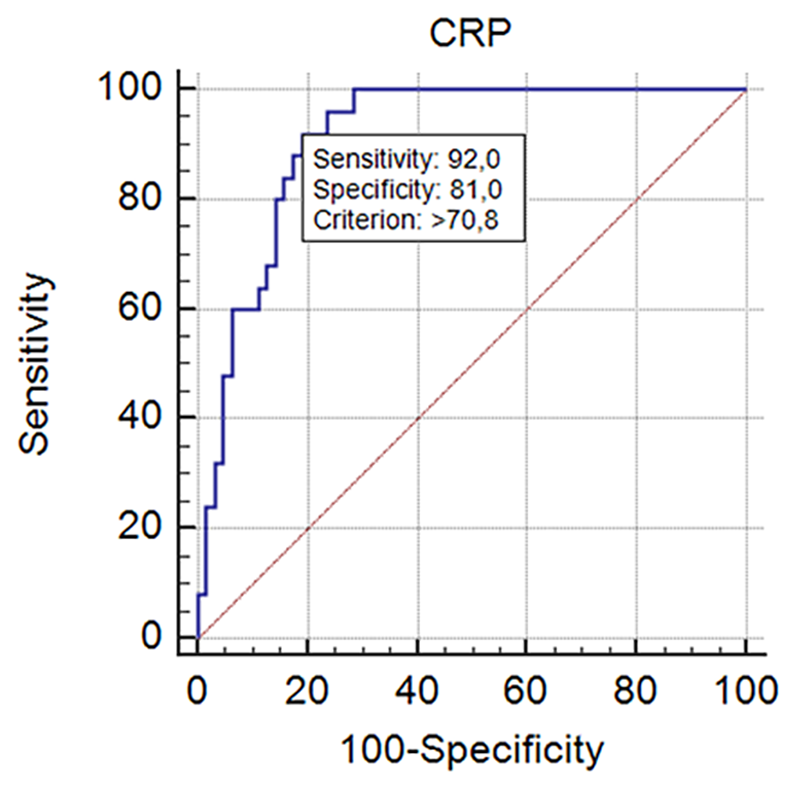

Supplement: S1 Fig — The area under the curve reaches 0.91 for a cut-off set at 70 mg/L, with a sensitivity of 92% and a specificity of 81%. (TIF) [file pone.0161277.s001.tif]
