# Supplementary material for: Diagnostic Algorithm in the Management of Acute Febrile Abdomen in Patients with Autosomal Dominant Polycystic Kidney Disease
Source: PLoS One. 2016 Aug 16;11(8):e0161277. doi: 10.1371/journal.pone.0161277 (PMC4987061; doi:10.1371/journal.pone.0161277)
Supplement: S1 File — (PDF) [file pone.0161277.s002.pdf]

	Age	Gender (0=F)	Dialysis	KTR	eGFR (ml/min)	T°>38°C	Pain	WBC (x1000/mm³)	CRP (mg/L)	Hematuria	Leucocyturia	Germ ID urines
CyH	51	0	0	0	32	0	1	7.1	25	0	0	0
CyH	45	0	0	0	51	0	1	8.44	1	1	0	0
CyH	44	1	0	1	98	0	0	10.84	1	1	1	1
CyH	42	1	0	0	56	0	1	11.11	21	0	0	0
CyH	41	1	0	0	145	0	1	6.8	1	1	0	0
CyH	42	1	0	0	150	0	1	15.7	11	0	0	0
CyH	42	1	0	0	126	0	1	7.5	2	0	0	0
CyH	45	1	0	0	151	0	1	5.37	2	0	0	0
CyH	45	1	0	0	155	0	1	13.23	28	0	0	0
CyH	56	0	0	1	92	0	1	9.85	62	1	1	1
CyH	39	1	0	1	50	0	1	6.8	25	1	1	0
CyH	66	0	0	1	23	0	1	11.67	34	0	0	0
CyH	55	0	0	0	56	0	1	6.42	16	1	0	1
CyH	66	1	0	0	19	1	1	15.72	1	0	0	0
CyH	81	0	0	0	28	0	1	14.18	10	0	0	0
CyH	49	0	0	0	47	0	1	11.29	12	0	0	0
CyH	38	0	0	1	38	0	1	5.32	1	0	0	0
CyH	55	0	0	0	28	0	1	7.77	13	0	0	0
CyH	32	1	0	0	157	0	1	12.99	5	0	0	0
CyH	36	1	0	0	123	0	1	14.64	7	0	0	0
CyH	50	0	0	0	44	0	1	13.1	6	0	0	0
CyH	47	1	0	0	134	0	1	13.45	1	0	0	0
CyH	63	1	0	0	21	0	1	10.17	38	1	0	0
CyH	63	1	0	0	19	0	1	8.29	8	0	0	0
CyH	26	1	0	0	43	0	1	9.58	14	0	0	0
CyH	27	1	0	0	19	0	1	9.88	5	0	0	0
CyH	31	0	0	0	96	0	1	4.86	24	0	0	0
CyH	32	0	0	0	111	0	1	5.87	3	0	0	0
CyH	33	0	0	0	107	0	1	7.16	24	0	0	0
CyH	35	0	0	0	100	1	1	5.83	1	0	0	0

Renal Cyl	37	1	0	0	12	1	1	8.32	99	1	1	0
Renal Cyl	61	1	1	0		1	1	7.49	63	0	0	0
Renal Cyl	38	1	0	1	52	1	1	11.7	204	0	0	0
Renal Cyl	56	0	0	1	34	1	0	10.74	119	0	0	0
Renal Cyl	68	0	0	0	56	1	1	19.26	305	0	1	1
Renal Cyl	54	1	0	0	46	1	1	11.7	78	0	0	0
Renal Cyl	50	1	0	1	37	1	1	10.05	273	1	1	0
Renal Cyl	56	0	0	0	62	1	1	8.34	206	0	0	0
Renal Cyl	60	0	0	1	22	1	1	9.21	251	1	1	1
Renal Cyl	19	0	0	0	124	1	1	13.26	127	0	0	0
Renal Cyl	53	0	1	0		1	1	8.27	703	1	1	1
Renal Cyl	54	0	0	1	45	1	1	10.84	328	1	1	1
Renal Cyl	55	0	0	1	49	1	1	17.4	217	0	1	1
Renal Cyl	36	1	0	0	15	1	1	6.46	11	0	0	0
Renal Cyl	51	1	1	0		1	1	11.68	133	0	0	0
Renal Cyl	33	1	0	0	129	1	1	10.23	79	0	0	0
Renal Cyl	65	0	0	0	41	1	1	12.24	463	1	0	0
Liver Cyl	66	1	1	1		1	1	9.53	150	0	0	0
Liver Cyl	66	1	0	1	13	1	1	12.17	173	0	1	0
Liver Cyl	66	0	0	0	36	1	1	11.05	341	0	0	0
Liver Cyl	56	0	0	1	39	1	1	15.58	256	1	0	0
Liver Cyl	66	0	0	1	38	1	1	4.25	51	0	0	0
Liver Cyl	66	1	1	1		1	1	6.5	342	0	0	0
IUO	69	0	0	1	44	1	1	6.28	15	1	1	0
IUO	65	0	0	1	36	1	0	2.82	28	1	0	0
IUO	63	0	0	1	40	0	0	7.71	132	0	1	1
IUO	49	0	0	1	135	0	0	11.52	39	1	0	1
IUO	37	1	0	0	19	0	1	4.37	32	0	0	0
IUO	32	1	0	1	12	0	0	22.97	151	0	0	1
IUO	46	1	0	1	42	1	0	12.47	250	1	1	0
IUO	32	1	0	0	142	1	1	11.96	38	0	0	0

IUO	76	0	0	1	34	0	0	13.07	253	0	1	0
IUO	64	0	0	1	53	0	0	13.41	214	1	1	0
IUO	78	1	0	1	28	1	0	11.14	145	0	0	0
IUO	68	0	0	1	45	0	1	5.8	17	0	0	1
IUO	30	0	0	0	97	0	1	9.93	47	0	1	1
IUO	31	0	0	0	56	1	1	18.1	386	0	1	1
IUO	56	0	0	1	43	1	1	11.94	163	0	1	0
IUO	49	0	0	0	37	0	1	10.35	108	1	1	1
IUO	59	1	0	0	19	0	1	10.4	68	0	0	0
IUO	57	1	0	1	80	1	0	13.5	25	0	1	0
IUO	64	1	0	1	55	1	1	18.23	6	1	1	1
IUO	58	1	0	1	48	1	1	21.12	53	1	1	1
IUO	24	0	0	0	85	1	1	5.22	89	0	0	0
IUO	48	1	HD	0	4	0	1	7.73	165	1	1	0
IUO	56	1	0	0	41	1	0	6.52	208	1	1	1
IUO	75	1	0	1	29	1	1	19.52	71	1	1	1
IUO	72	1	0	1	38	1	1	12.29	26	0	0	0
IUO	57	0	0	1	31	1	1	9.93	30	0	1	0
IUO	58	0	0	1	30	1	1	7.64	78	0	0	0
IUO	55	0	0	1	36	1	1	9.2	50	0	1	1
IUO	35	1	DP	0		0	1	8.45	22	1	0	0
IUO	48	1	0	1	48	1	1	14.6	66	1	1	1
IUO	47	1	0	1	21	1	1	10.62	50	0	1	1
IUO	65	0	0	0	51	1	1	24.34	17	0	0	0
IUO	69	0	0	1	26	1	1	6.81	42	0	0	1
IUO	84	0	0	0	17	1	0	23.92	278	0	1	1
IUO	44	1	0	1	88	1	0	10.48	60	0	1	1

Germ Id urine	Germ ID blood	Germ ID blood	PET/CT
	0		
	0		
K. Pneumoniae	1	K. Pneumoniae	
	0		
	0		
	0		
	0		
	0		
	0		
E.coli	1	S.epidermidis	
	0		negative
	0		
S. agalactiae	0		
	0		
	0		
	0		
	0		
	0		
	0		
	0		
	0		
	0		
	0		
	0		
	0		
	0		
	0		
	0		
	0		
	0		

	1	S.capitis	positive
	0		
	0		
	1	E.coli	
E.coli	1	S.epidermidis	
	0		positive
	1	E.coli	positive
	0		
E.coli	0		
	1	E coli	
E.coli	1	Ecoli	
E.coli	0		positive
E.coli	0		
	0		positive
	0		positive
	0		
	0		negative
	1	E.coli	
	0		
	0		positive
	1	E.coli	
	0		negative
	1	E.coli, E.durans	positive
	0		
	0		negative
E.coli	1	S.epidermidis	negative
E.coli	0		
	0		
P.mirabilis	0		
	0		positive
	0		

	0		
	0		positive
	0		
E.coli	0		
C.koseri	0		
E.coli	0		
	1	E.coli	negative
E.coli	0		
	0		
	0		positive (other)
E.coli	0		negative
E.coli	1	E.coli	
	0		
	0		negative
S.aureus	1	S.aureus	
E.fecalis	0		negative
	1	K.pneumoniae	positive (other)
	0		negative
	0		negative
K.pneumoniae	0		positive (other)
	0		negative
E.coli	1	E.coli	
E.coli	1	E.coli	positive (other)
	0		
E.coli	0		positive (other)
E.coli	0		
K.pneumoniae	1	K.pneumoniae	negative
