# Supplementary material for: Diagnostic Algorithm in the Management of Acute Febrile Abdomen in Patients with Autosomal Dominant Polycystic Kidney Disease
Source: PLoS One. 2016 Aug 16;11(8):e0161277. doi: 10.1371/journal.pone.0161277 (PMC4987061; doi:10.1371/journal.pone.0161277)
Supplement: S2 File — (PDF) [file pone.0161277.s003.pdf]

Detailed characteristics of the cohort including 53 ADPKD patients with 88 events of suspected cyst complication

Patients with one type of cyst complication:

- 9 patients had 1 cyst hemorrhage
- 5 patients had 1 renal cyst infection
- 18 patients had 1 IUO

Patients with several times the same type of cyst complication:

- 1 patient had 5 cyst hemorrhages
- 1 patient had 4 cyst hemorrhages
- 3 patients had 2 cyst hemorrhages
- 4 patients had 2 IUO
- 1 patient with 3 liver cyst infections

Patients with different types of cyst complications:

- 1 patient with 1 cyst hemorrhage and 1 IUO
- 1 patient with 1 cyst hemorrhage and 3 renal cyst infection
- 1 patient with 1 cyst hemorrhage, 1 IUO and 2 renal cyst infections
- 1 patient with 1 cyst hemorrhage and 1 renal cyst infection
- 1 patient with 2 cyst hemorrhages, 1 IUO and 1 renal cyst infection
- 2 patients with 1 IUO and 1 renal cyst infection
- 1 patient with 1 IUO and 1 renal cyst infection
- 2 patients with 1 IUO, 1 hepatic cyst infection and 1 renal cyst infection
- 1 patient with 1 IUO and 1 liver cyst infection
